# Evaluation of deprescription by general practitioners in elderly people with different levels of dependence: cross-sectional study

**DOI:** 10.1186/s12875-024-02299-3

**Published:** 2024-03-02

**Authors:** Tânia Coelho, Inês Rosendo, Carlos Seiça Cardoso

**Affiliations:** 1 Unidade de Saúde Familiar VitaSaurium, Soure, Coimbra Portugal; 2 CINTESIS - Center for Health Technology and Services Research; Faculty of Medicine, University of Porto, Porto, Portugal; 3 Unidade de Saúde Familiar Coimbra Centro, Coimbra, Portugal; 4 Unidade de Saúde Familiar Condeixa, Condeixa, Portugal; 5 Faculty of Medicine, University of Coimbra, Coimbra, Portugal

**Keywords:** Deprescribing, Aged, Dependence, General practice, Polymedication

## Abstract

**Background:**

Polypharmacy is easily achieved in elderly patients with multimorbidity and it is associated with a higher risk of potentially inappropriate medication use and worse health outcomes. Studies have shown that deprescription is safe, however, some barriers have been identified. The aim of this study was to analyse Portuguese General Practitioners (GP) deprescription’s attitudes using clinical vignettes.

**Methods:**

Cross-sectional study using an online survey with 3 sections: demographic and professional characterization; two clinical vignettes with an elderly patient with multimorbidity and polypharmacy in which the dependency level varies; barriers and factors influencing deprescription. Frequencies, means, and standard deviations were calculated to describe the GPs. Analysis of the deprescription attitude, globally and for each drug, for each clinical vignette applying the McNeemar’s test.

**Results:**

A sample of 396 GP was obtained with a mean age of 38 years, most of them female. A statistically significant difference (*p* < 0.01) was observed in deprescribing according to the patient dependency level, with more GPs (80.4% versus 75.3%) deprescribing in the most dependent patient. A statistically significant difference was found for all drugs except for antihypertensive drugs. All medications were deprescribed more often in dependent patients except for anti-dementia drugs. More than 70% of the participants considered life expectancy and quality of life as “very important” factors for deprescription and more than 90% classified the existence of guidelines and the risks and benefits of medication as “very important” or “important”. In the open question, the factors most reported by the GP were those related to the patient (52,9%).

**Conclusions:**

This is the largest study on this topic carried out in Portugal using clinical vignettes, with a representative sample of Portuguese GP. The level of dependence significatively influenced the deprescription attitude of Portuguese GPs. The majority of the GPs classified the quality of life, life expectancies, potential negative effects and the existence of guidelines as “very important” or “important” while deprescribing. It is important to develop and test deprescribing in real life studies to analyze if these attitudes are the same in daily practice.

**Supplementary Information:**

The online version contains supplementary material available at 10.1186/s12875-024-02299-3.

## Background

Polymedication is defined as the simultaneous use of five or more drugs, and it may lead to use of inappropriate/inefficient medication or therapeutic duplication [1–3]. It is easily achieved, in the light of current guidelines, in elderly patients with multimorbidity (with three or more chronic conditions) [1, 4, 5], being estimated that 30–70% of elderly patients are polymedicated [6]. Data from Portugal points to 80% of elderly people having multimorbidity [7] and 70% being polymedicated [8]. 

The prescription cascade, know as an adverse effect of a drug is misinterpreted as a new symptom, leading to the prescription of more medication [3, 9, 10], is also a main factor contributing to polymedication [11]. 

Polimedication is related to poorer quality of life [5] and worse health outcomes [9, 12], including falls, fractures, hospitalizations, cognitive changes [2], institutionalization, and greater likelihood of poor adherence to therapy [3], adverse reactions [13], drug interactions, and medical error [4, 6]. There seems to be an association between polymedication and mortality [9], although there is no consensus on whether polymedication is a marker of poorer health status or an independent risk factor for mortality [14]. Polymedication also contributes to increased health care costs [3], both directly and indirectly (hospitalizations, emergency room visits, or consultations).

Potentially inappropriate medication is defined as medication whose risks outweigh the benefits, which is not suitable for the patient’s objectives or whose efficacy is not established [6] when alternatives of equal or greater efficacy [15] are available. It is estimated to be present in about 40–60% of elderly patients, and besides this percentage varies between studies [15–18], it is more likely to be found in polymedicated patients [3]. 

Deprescribing is the act of reducing the number of prescribed drugs, reducing the dose of a given medication [19], replacing it with a safer medication, or decreasing the intake frequency in a supervised manner, to discontinue medication that is not aligned with the therapeutic goals to improve patients’ health outcomes [3, 10, 12, 20]. It is not without risks, and may be associated with weaning-related symptoms, disease relapse, and pharmacokinetic/pharmacodynamic changes in the remaining drugs [6], however, studies have shown that deprescribing is feasible and safe if performed according to the recommendations [16]. Despite this, barriers to deprescribing have been identified, either related to the patient and family - fear of worsening disease or adverse effects, lack of alternative medication, lack of family support, bad experiences in the past, feeling that the doctor is giving up, cognitive changes [16] - or related to the physician - lack of guidance standards, fear of symptoms upon discontinuation or relapse of the disease, difficulty in discussing life expectancy with the patient, and high time consumption [1, 3, 10]. 

GPs are in the ideal position to prescribe [19], in an individualized manner [21] and based on the doctor-patient relationship previously established, which has been shown to contribute to the patient’s confidence in the deprescribing process [16]. 

Two studies regarding deprescription in the elderly with multimorbidity were carried out using clinical vignettes. In both of them, the presence of cardiovascular disease (CVD) [1] was varied, and in one of them, the degree of dependence of the patient was also varied [22]. The latter was carried out in Primary Health Care in Portugal, and to our knowledge, it is the only study of this scope carried out in this setting in Portugal that included 285 participants. In this study, three levels of dependence, low, medium, and high, were used, with more physicians deprescribing in patients with high dependence (90.2%) and fewer in patients with low dependence (75.1%).

### Objective

The main objective of this study is to characterize the deprescribing attitudes of GPs in Portugal towards elderly patients with multimorbidity and polymedication, in which the level of dependence varies, using clinical vignettes. The secondary objective is to identify the main barriers to deprescribing identified by the same GPs.

## Methods

### Study design

Cross-sectional study conducted through the application of online questionnaires using the google forms® platform. Approved by the Ethics Committee of the Faculty of Medicine of the University of Coimbra.

### Participants

Recruited through mailing lists, institutional e-mails of the Family Health Units (FHU) and Personalized Health Care Units (PHCU) of Mainland Portugal and institutional e-mails of the professionals of ARS Centro[Regional Health Authority-hereinafter RHA], aiming to obtain a minimum of 377 responses, calculated as the representative sample of the GPs in Portugal, using the site http://www.raosoft.com/samplesize.html, for a confidence interval (CI) of 95%.

### Questionnaire

Prior to the presentation of the questionnaire, each participant was asked to consent to its completion, with guaranteed anonymity regarding the answers.

The questionnaire was adapted from the LESS [1] study and was composed of three sections: (a) sociodemographic information, (b) clinical vignette, and (c) barriers and factors that encourage deprescription (Annex 1).

In section a) not only the participant’s demographic information was collected, but also their professional information, namely, age, gender, RHA where they work, type of health unit, number of years they have been practicing General and Family Medicine (GFM) (including internship years and excluding the current year) and professional category (intern or specialist). Also, in this section, the physician was asked about the number of consultations per day (including telephone and face-to-face consultations and excluding non-face-to-face consultations), how often the physician consulted elderly patients with multimorbidity and polymedication, and how often the physician dealt with the topic of deprescribing and effectively deprescribed patients with these characteristics.

The second section, b), was composed of two clinical vignettes concerning a hypothetical elderly patient with multimorbidity and polymedication: an 80-year-old man with a history of ischemic heart disease (stent placed 15 years ago), hypercholesterolemia (total cholesterol of 220 mg/dL in the last analyses), hypertension (160/80mmHg in the last consultations), arthrosis of the knees and a tendency to constipation. He was taking Donepezil 10 mg daily, Acetylsalicylic acid (ASA) 100 mg daily, Atorvastatin 40 mg daily, Ramipril 5 mg daily, Amlodipine 5 mg daily, Sene 2 pills at night, Paracetamol 1 g 4 times a day, Tramadol 50 mg 3 times a day. He had no renal, liver or thyroid function changes. Between the two vignettes the dependency level of this patient varied: in the first vignette the patient was dependent for activities of daily living (ADLs) and with Dementia, scoring 18/30 on the Mini-Mental State Examination (MMSE); in the second, the patient was fully autonomous for ADLs and had only mild cognitive changes, scoring 23/30 on the MMSE. The clinical vignettes were also adapted from the LESS [1]. 

In the last section, c), participants were asked to categorize from “not at all important” to “very important” when deprescribing two sets of criteria, adapted from the LESS study [1], one related to the patient (age, life expectancy, quality of life, previous experiences, expectations, potential negative effects, communication difficulties, and family expectations) and one related to the physician/clinical practice (existence of guidelines and deprescribing tools, communication and collaboration with physicians from other specialties, time consumption, benefit and risks of medication). Finally, two open-ended, optional questions were asked about other factors that may influence deprescribing and additional comments to the study.

### Statistical analysis

Frequencies, means and standard deviations (SD) were calculated to describe the demographic and professional characterization of the general practitioners surveyed.

McNeemar’s test was used to compare the deprescription in the case of the most dependent patient versus the case of the most autonomous patient, in total and per medication.

The open-ended answers to the question about other factors that might influence deprescription were subjected to content analysis and classified according to the categories defined by Anderson et al. in their systematic review on barriers and facilitators of deprescription: [23] “conscience” of the prescriber about his prescription; “inertia” divided into “beliefs and attitudes” of the general practitioner about the consequences that might result from deprescription, and “behaviour” of the general practitioner with respect to whether or not he/she takes responsibility for prescribing; “Self-efficacy” divided into the “skills and knowledge” of the GP (experience, training) and “information/influence” namely from guidelines, literature or other specialists; “Feasibility” related to factors external to the GP that may be related to the “patient” (resistance to change), the “resources” (time, effort, psychosocial support), the “clinical practice” of renewing prescriptions without reviewing them, the “medical culture” that encourages respect for the autonomy of the initial prescriber, the “health culture, and beliefs” that encourage prescribing as a way to validate the disease and “regulatory” issues related to measures to assess the quality of clinical practice based on quantitative aspects guided by clinical guidelines.

For quantitative analysis SPSSv28® was used and for qualitative analysis MaxQda 2022®.

## Results

### A) Demographic and professional characterization of the sample

The study was answered by 396 GPs, with a mean age of 38 years (minimum 25, maximum 69; SD 10,262), mostly female (76.5%), and specialized in GFM (75.3%). The same number of participants, 129, belonged to the Central and Northern RHA (32.6%), with a very similar number of participants, 126, belonging to the Lisbon and Tejo Valley RHA (31.6%) and only a small minority belonging to the Alentejo (0.8%) or Algarve (2%) RHA. More than half of the participants were working in FHU model B (51.8%), 112 worked in FHU model A (28.3%) and the remaining ones in Personalized Health Care Units (PHCU) (19.4%) or elsewhere (0.5%). On average, participants had been practicing GFM for 11 years (minimum 0, maximum 43; SD 9.83) (Table 1).

**Table 1:** Demographic and professional characterization

Characterization of participants (*n* = 396)	
Age, Mean (SD)	38,48 (10,262)
Years of GFM practice, mean (SD)	11,07 (9,830)
Gender • Feminine • Masculine	n (%) 303 (76,5) 93 (23,5)
Professional Category	
• Intern • Specialist	98 (24,7) 298 (75,3)
RHA	
• Alentejo • Algarve • Centre • Lisboa and tejo valley • North • Elsewhere	3 (0,8) 8 (2) 129 (32,6) 126 (31,6) 128 (32,6) 1 (0,3)
Typology of the unit	
• FHU • Other	394 (99,5) 2 (0,5)

Legend: FHU – Family Health Unit

Most of the general practitioners surveyed performed between 15 and 25 consultations per day (60.9%), 19.4% performed between 26 and 35 consultations per day, 14.9% performed fewer than 15, and 4.8% performed more than 35. When asked how often they performed consultations for elderly patients with multimorbidity and polymedication, none of the participants classified this frequency as “never” or “rarely”. About half of the general practitioners reported seeing patients with these characteristics “very often” (51.8%) or “frequently” (46%) and only 9% considered doing it “occasionally”. Still regarding patients with these characteristics, 44.7% of general practitioners assume they deal with the issue of deprescribing “frequently”, but only 26% deprescribe “frequently”, and most of the participants describe it only “occasionally” (55,6%) (Table 2).

**Table 2:** Characterization of clinical activity and deprescription attitude

Characterization of clinical activity	
Consultations per day • < 15 • 15–25 • 26–35 • > 35	n (%) 59 (14.9) 251 (60.9) 77 (19.4) 19 (4.8)
Consultation with an elderly with multimorbidity and polymedication	
• Never • Rarely • Occasionally • Often • Very frequently	0 0 9 (2.3) 182 (46) 205 (51.8)
Dealing with deprescription	
• Never • Rarely • Occasionally • Often • Very frequently	1 (0.3) 18 (4.5) 136 (34.3) 177 (44.7) 64 (16.2)
Deprescribing	
• Never • Rarely • Occasionally • Often • Very frequently	0 49 (12.4) 220 (55.6) 103 (26) 24 (6.1)

### B) Clinical vignettes: deprescription according to the degree of dependence

Comparing the clinical vignette in which the patient has a higher degree of dependence with the one in which the patient is more autonomous, there is a statistically significant difference (*p* < 0.01) between the number of general practitioners who would deprescribe at least one medication, with more general practitioners deprescribing in the dependent patient (88.4%) (Table 3). On average, participants would deprescribe 2,18 drugs in the dependent patient and 1,66 drugs in the more autonomous patient.

**Table 3:** Comparison of the percentages of GPs who deprescribed according to dependency level

	Dependent patient	Autonomous patient	p^1^ value
% de GP that would deprescribe (mean^2^ ± SD)	88.4% (2.18 ± 1.33)	75.3% (1.66 ± 1.44)	< 0.01

Legend: *p* value^1^ : McNeemar’s test; ^﻿2^mean; mean number of deprescribed drugs. GP – General Practitioners

Comparing now the percentage of GPs who would deprescribe each of the drugs for the clinical vignette of the dependent patient for ADLs versus the clinical vignette of the autonomous patient for ADLs, it can be seen that there is a statistically significant difference for the following drugs: Donepezil (*p* < 0.01), ASA (*p* < 0.01), Atorvastatin (*p* < 0.01), Sene (*p* < 0.01), Paracetamol (*p* < 0.01), and Tramadol (*p* < 0.01). Among these, all were deprescribed by more GPs in the dependent patient, except Donepezil which was deprescribed more often in the autonomous patient.

Of all the drugs under analysis, the ones more subject to being deprescribed, in both patients, were the analgesics Tramadol and Paracetamol, with 58% and 42.7% of the GPs deprescribing Tramadol and 54.5% and 39.5% deprescribing Paracetamol, in the dependent and autonomous patient, respectively.

For the antihypertensives studied, no statistically significant difference was observed, and they were the drugs deprescribed by the fewest general practitioners, only about 3% in each case (Table 4).

**Table 4:** Comparison of the percentages of general practitioners who have deprescribed, by drug, according to the level of dependence

Drugs	Dependent patient % (CI 95%)	Autonomous patient% (CI 95%)	p^1^ value
Donepezil 10 mg id	11.4% (8,5 a 14,7)	32.1% (27,6 a 36,8)	< 0.01
AAS 100 mg id	17.9% (14,4 a 21,9)	8.3% (5,9 a 11,3)	< 0.01
Atorvastatin 40 mg id	33.8% (29,3 a 38,6)	14.4% (11,2 a 18,1)	< 0.01
Ramipril 5 mg id	3.3% (1,8 a 5,4)	3.0% (1,6 a 5,0)	1.00
Amlodipine 5 mg id	3.5% (2,0 a 5,7)	3.0% (1,6 a 5,0)	0.625
Sene 2 tablets id	35.4% (30,8 a 40,1)	24.2% (20,2 a 28,6)	< 0.01
Paracetamol 1 g 4id	54.5% (49,6 a 59,4)	39.4% (34,7 a 44,3)	< 0.01
Tramadol 50 4id	58% (52,1 a 62,8)	42.7% (37,9 a 47,6)	< 0.01

Legend: ^1^*p*-value: McNeemar’s test, id – daily

### C) Barriers and potentiating factors of deprescription

With regard to the barriers and factors that promote deprescription, most general practitioners considered all patient-related factors presented as “important” or “very important”. More than 70% of the physicians considered life expectancy (71.7%) and quality of life (75.8%) as “very important” characteristics to consider when deprescribing. Family expectations (19.2%) and previous experiences with deprescribing (15.9%), were the factors that most GPs considered as neutral. Among the hypotheses presented, the one that more GPs (*n* = 29) classified as “not important” or “not very important” was age (7.4%) (Table 5).

**Table 5:** Classification of patient-related criteria by the GPs

Patient-related factors	Not at all important GP, n (%)	Not very important GP, n (%)	Neutral GP, n (%)	Important GP, n (%)	Very important GP, n (%)
Age	3 (0.8)	26 (6.6)	32 (8.1)	195 (49.2)	140 (35.4)
Life expectancy	1 (0.3)	3 (0.8)	11 (2.8)	97 (24.5)	384 (71.7)
Quality of Life	0	3 (0.8)	5 (1.3)	88 (22.2)	300 (75.8)
Previous experiences with deprescription	2 (0.5)	14 (3.5)	63 (15.9)	226 (57.1)	91 (23)
Patient expectations	1 (0.3)	2 (0.5)	25 (6.3)	224 (56.6)	144 (36.4)
Potential negative effects	0	2 (0.5)	12 (3)	140 (35.4)	242 (61.1)
Communication difficulties	3 (0.8)	14 (3.5)	43 (10.9)	214 (54)	122 (30.8)
Family expectations	2 (0.5)	18 (4.6)	76 (19.2)	230 (58.1)	70 (17.7)

Table 6 shows the results regarding factors related to the GP and clinical practice, similarly to those presented in Table 5 for the patient. The majority of the GPs again considered all the factors presented as “important” or “very important”, with more than 90% of the physicians considering one of these classifications for the existence of guidelines (90.2%), the benefit of medication (97.8%) and the risks of medication (98.5%). Medication risks were also the factor that more physicians (*n* = 265) considered as “very important” (66.9%). Time consumption was the topic that divided physicians the most with 43.2% of physicians rating it as “important” and about a fifth as “neutral” (20.5%) or “very important” (25.5%), but with few physicians rating it as “not at all important” (2%) or “not very important” (8.8%). All other factors were rated as “not at all important” or “not very important” by less than 5% of physicians.

**Table 6:** Classification by the GPs of the criteria related to the physician/clinical practice

GP/clinical practice related factors	Not at all important GP, n (%)	Not very important GP, n (%)	Neutral GP, n (%)	Important GP, n (%)	Very important GP, n (%)
Existence of deprescribing guidelines	3 (0.8)	9 (2.3)	27 (6.8)	198 (50)	159 (40.2)
Existence of tools to facilitate deprescribing	5 (1.3)	8 (2.0)	33 (8.3)	196 (49.5)	154 (38.9)
Communication with physicians from other specialties	1 (0.3)	10 (2.5)	48 (12.1)	208 (52.5)	129 (32.6)
Collaboration with physicians from other specialties	1 (0.3)	9 (2.3)	52 (13.4)	202 (51)	131 (33.1)
Time Consumption	8 (2.0)	35 (8.8)	81 (20.5)	171 (43.2)	101 (25.5)
Benefit of medication	0	2 (0.5)	7 (1.8)	165 (41.7)	222 (56.1)
Medication risks	0	3 (0.8)	3 (0.8)	125 (31.6)	265 (66.9)

Legend: GP – General Practitioners

38.4% of the GPs [33.7–43.2%; 95%CI] consider that there are other factors that can influence deprescribing and 30.8% of the respondents answered the optional open question indicating which factors. From the 122 responses, we obtained 138 codes (Table 7).

More than half (52.9%) of the GPs identified factors related to the user, namely their will and acceptance and that of their family members, therapeutic adherence, health literacy, and economic aspects. Twenty participants (14.49%) identified resources as an important factor for deprescribing, particularly the consultation time and the social and family support available to the patient. The physician’s beliefs and attitude were also identified as important by 12.32% of the physicians, regarding what they consider to be beneficial/harmful effects, adverse effects or intolerance to medication, and also their fear of deprescription. None of the participants mentioned factors related to health culture, medical culture, and clinical practice as influential to deprescription in this open question.

**Table 7:** Responses to the open-ended question about factors that might influence deprescription

Categories		Examples	GP, n (%)
Conscience		• “Making life easier for the patient who has less medication to take” • “Sensitivity of the impact of each drug on quality of life”	2 (1.45)
Inertia	Attitudes and Beliefs	• “Adverse effects” • “Benefit-risk of the drug” • “Medication intolerance” • “Psychological effect of deprescription on the patient” • “Pharmacodynamics and pharmacokinetics” • “Fears and beliefs of the physician; defensive medicine”	17 (12.32)
	Behaviour	• “Who prescribed it (GFM versus hospital)” • “Withdrawing medication prescribed by other doctors a long time ago (especially privately)”	2 (1.45)
Self-efficacy	Information/ Influence	• “Non-medical advertising about the medication” • “Association of the drug with the physician who prescribed it, who was very good” • “Lack of communication between specialties” • Non-existence of guidelines • “Opinion of other physicians who follow the patient”	8 (5.8)
	Capabilities/ Knowledge	• “Physician’s prior knowledge” • “Prior experience with deprescription” • “Unawareness” • “Professional training”	11 (7.97)
Feasibility	Regulation	• “Factors related to clinical governance and safety culture”	1 (0.72)
	Health culture		0
	Medical Culture		0
	Clinical practice		0
	Resources (psychosocial support)	• “Family/social support • “Existence of alternatives” • “Feedback from pharmacies” • “Other priority actions” • “Consultation time”	20 (14.49)
	Patient (will, preference, etc.)	• “The patient’s adhesion” • The patient’s willingness • Acceptance of the patient/relatives • “Background, degree of dependence, frailty” • Patient’s beliefs • Literacy • Economic aspects”	73 (52.9)
Non codable		• “Difficulty in effectively controlling complaints” • “Control test results; controlled diet” • “Changes in habits of the user that no longer justify taking the medication” • “Anxiety disorders/psychiatric illness in general”	4 (2.9)
Total			138

## Discussion

From the previous literature search, in a 2021 study based on clinical vignettes of patients over 80 years of age, observed that the likelihood of deprescribing was higher in patients with a high level of dependence and cognitive changes [24], parallel to our results.

In the previous study conducted in Portugal, the level of dependence directly influenced the deprescription for all the studied drugs including antihypertensive drugs [22]. This was not the case in our study, in which the level of dependence did not influence the deprescription of Ramipril (*p* = 1) and Amlodipine (*p* = 0.625). Ouer results are aligned with literature data that demonstrates that the likelihood of deprescribing antihypertensive medication in elderly patients with hypertension was low, even for blood pressure values below therapeutic targets probably because the harms of overmedication are rarely addressed in clinical guidelines [25]. In the elderly over 80 years of age, antihypertensive medications are among those least likely to be deprescribed [24], however, frailty is associated with the decision not to initiate antihypertensive medication, probably because higher systolic blood pressure is considered protective of overall mortality risk in this population [26]. 

The deprescription of statins, even in the case of tertiary prevention, is acceptable and safe in a patient with low life expectancy [27], since cardiovascular prevention is no longer a priority [28]. However, the main reason for deprescription of cardiovascular preventive medication is suspected adverse reaction [29] and statins are the least deprescribed class of preventive drugs in patients with low life expectancy [30]. Despite this literature data, we found a a statistically significant difference in deprescription of statin and ASA with more GPs deprescribing in the dependent patient.

Although the literature is controversial regarding the use of Donepezil in patients with severe dementia, it has been shown to have modest cognitive and functional benefits in patients with moderate Alzheimer’s disease [31]. In our study, donepezil was deprescribed by fewer GPs in the dependent patient, with statistically significant difference, probably because the dependent patient also had a diagnosis of dementia (MMSE score of 16/30).

The drugs deprescribed by a higher percentage of GPs in both clinical vignettes were Paracetamol and Tramadol, deprescribed by more than half of the sample in the dependent patient (54.5% and 58%) and by 39.4% and 42.7% of the physicians in the autonomous patient, respectively (*p* < 0.01). This is aligned with literature data that shows that physicians have greater difficulty in deprescribing preventive medication compared to symptomatic medications [4], and analgesic medications are the most deprescribed by GPs [24]. Although pain is a very prevalent symptom with aging, it is often underestimated and consequently inefficiently treated by physicians.

In the study on deprescription in primary health care in Portugal the Paracetamol dose of 1 gram 3 times a day was used and obtained deprescription values ranging from 32.6 to 38.6%, so the frequency of administration of 4id can justify the higher frequencies of deprescription observed by us [22]. 

On the other hand, the use of opioids in chronic pain patients should be titrated to an effective dose with the least possible adverse effects, and most adverse effects tend to decrease over time with the exception of constipation, a frequent cause of discontinuation of this therapy [32]. Two reasons can be given for the high rates of deprescription of Tramadol in both clinical vignettes: the tendency for patient constipation (a frequent side effect of opioid use) and the fact that Portugal is one of the countries with one of the lowest prescriptions of opioids in Europe, probably due to cultural, educational and economic issues [33]. 

With regard to patient-related barriers and triggers for deprescription, our data is in partial agreement with the LESS study, where Swiss GPs value the quality of life and potential negative effects in a similar way to Portuguese GPs. Swiss GPs don´t value as much as Portuguese GPs the potential negative effects and patient expectations [1]. In the aspects related to the physician/clinical practice, our results were similar to the LESS study were 99% and 98% of participants attributed importance (important or very important) to the risks and benefits of medication. There was a difference in the value that physicians attributed to the existence of guidelines on deprescription, wich was more valued by Portuguese GPs compared to the Swiss GPs [1]. GPs from the 31 countries rated quality of life (96%), life expectancy (90%), risk of adverse effects (94%), and the risks (98%) and benefits (95%) of medication as “very important” or “important” [24]. 

The differences observed in the responses of physicians from various countries may be related to existing cultural differences and even to possible differences in clinical practice.

### Strengths and limitations

This is the largest study carried out in Portugal on this topic, using clinical vignettes, and obtained the calculated sample (396 participants). It is an innovating work in an emerging subject, that followed a methodology previously applied and published in the literature.

The principal limitation is fact that this is a work based on clinical vignettes, since the clinical vignette can be simple and reductive, as referred to in the LESS study [1]. In addition, we asked the GPs if they would deprescribe or decrease the dose of any of the medications, not differentiating whether they would choose to stop the medication completely or just decrease the dose or frequency of administration.

The response to the vignette case may not traduce the attitude of the physician in real life practice, therefore, this study may lead to real life studies.

We admit the presence of selection bias whereby younger physicians responded in greater numbers (mean age of participants 38 years compared to a national median of [61–65] years) because of the probable greater facility with computerized means, and there may also have been a response bias whereby people more motivated towards this subject responded in greater numbers.

## Conclusion

The level of dependence influences the deprescription attitudes of the GPs, with a higher percentage of physicians deprescribing in the most dependent patient.

This study can serve as a starting point for research in this area, namely about the differences in the deprescription and initiation of medication according to the level of dependence. Despite increasing the level of complexity of the study, it would be interesting to conduct a real-life study to assess whether the attitudes of deprescription are a study finding or if they are effectively translated into the clinical practice of GPs.

### Electronic supplementary material

Below is the link to the electronic supplementary material.

Supplementary Material 1

## Data Availability

All data generated or analysed during this study are included in this published article [and its supplementary information files].

## Abbreviations

ASA: Acetylsalicylic acid
ARS: Administração Regional de Saúde [RHA]
ADLs: Activities of daily living
CVD: Cardiovascular disease
SD: Standard deviations
CI: Confidence interval
GP: General practitioners
GFM: General and Family Medicine
MMSE: Mini Mental State Examination
PHCU: Personalized Health Care Units
FHU: Family Health Units
