# Supplementary material for: Evaluation of deprescription by general practitioners in elderly people with different levels of dependence: cross-sectional study
Source: BMC Prim Care. 2024 Mar 2;25:78. doi: 10.1186/s12875-024-02299-3 (PMC10908147; doi:10.1186/s12875-024-02299-3)
Supplement: Supplementary file 1 — Supplementary Material 1 [file 12875_2024_2299_MOESM1_ESM.docx]

**Attachments**

**Annex 1:** Questionnaire used in the study

A) Demographic Information

1. indicate the RHA (Regional Health Administration) where you practice your profession.

- RHA North
- RHA Centre
- RHA Lisbon and Tejo Valley
- RHA Alentejo
- RHA Algarve

1. Indicate the typology of your unit.

- Family Health Unit
- Other

1. Please indicate your age (in years).
2. Select your gender:

- Masculine
- Feminine

1. Please indicate the number of years you have been practicing family medicine (including internship years and excluding the current year):
2. Indicate your professional category

- Intern in Family Medicine
- Specialist in Family Medicine

1. Please indicate the average number of consultations per day (including telephone and face-to-face consultations; excluding non-face-to-face activity such as prescription renewal)

- <15
- 15-25
- 26-35
- >35

1. How often do you see patients who meet the following criteria: Age ≥65 years; Pluripathology (≥3 chronic pathologies); Polymedication (≥ 5 drugs belonging to the usual medication).

- Never
- Rarely
- Occasionally
- Frequently
- Very frequently

1. How often do you deal with the topic of deprescription in your daily clinical practice in patients with the characteristics described in question 8 (age ≥65 years, pluripatology and polymedication)?

- Never
- Rarely
- Occasionally
- Frequently
- Very frequently

1. How often do you deprescribe drugs in your daily practice consultations for patients with the characteristics described in question 8 (age ≥65 years, pluripathology and polymedication)?

- Never
- Rarely
- Occasionally
- Frequently
- Very frequently

B) Clinical case:

Patient X, male, 80 years old.

Social Background: retired, previously a train driver. Does not drive.

Medical history: Presented to consultation for dementia, presumably Alzheimer's disease. Has become more dependent in the last year, requiring help with household chores and with changing clothes. Scores 16/30 on the MMSE (scored 18/30 3 months ago).

Personal Background: Ischemic heart disease (a stent was placed 15 years ago, since then without angina), hypercholesterolemia and hypertension (with values of 160/80 mmHg in the last two consultations), arthrosis of the knees (causes morning pain) and tendency to constipation.

Additional diagnostic tests: total cholesterol of 220.4mg/dl, blood count is unchanged, kidney, liver and thyroid functions are unchanged, total colonoscopy at age 70 is unchanged, CT-CE with evidence of microvascular disease.

Usual Medication: Donepezil 19 mg id, Aspirin 100mg id, Atorvastatin 40 mg id, Ramipril 5mg id, Amlodipine 5mg id, Sene 2 tablets at night, Paracetamol 1g 4id, Tramadol 50 4id

1. Would you deprescribe any medication or decrease the dosage of any/any of the medications?

- Yes
- No

1. Which drug(s) would you deprescribe or lower the dose?

- Donepezil 19 mg id
- Aspirin 100mg id
- Atorvastatin 40 mg id
- Ramipril 5mg id
- Amlodipine 5mg id
- Sene 2 tablets evening
- Paracetamol 1g 4id
- Tramadol 50 4id

1. Now suppose that this patient had only mild cognitive changes, was fully independent for activities of daily living, and had an MMSE score of 23/30. Would you consider deprescribing or reducing the dose of any of the previously mentioned medications?

- Yes
- No

1. Which drug(s) would you deprescribe or lower the dose, given the scenario previously described in question 13?

- Donepezil 19 mg id
- Aspirin 100mg id
- Atorvastatin 40mg id
- Ramipril 5mg id
- Amlodipine 5mg id
- Sene 2 tablets evening
- Paracetamol 1g 4id
- Tramadol 50 4id

C) Barriers and potentiating factors of deprescription

1. How important are the following patient characteristics when considering deprescribing a particular medication?

|  | Not at all important | Not very important | Neutral | Important | Very important |
| --- | --- | --- | --- | --- | --- |
| Age |  |  |  |  |  |
| Life expectancy |  |  |  |  |  |
| Quality of life |  |  |  |  |  |
| Previous experiences with deprescribing |  |  |  |  |  |
| Patient expectations |  |  |  |  |  |
| Potential negative effects |  |  |  |  |  |
| Communication difficulties |  |  |  |  |  |
| Family expectations |  |  |  |  |  |

1. How important are the following criteria when you are considering deprescribing medication?

|  | Not important | Not very important | Neutral | Important | Very important |
| --- | --- | --- | --- | --- | --- |
| Existence of deprescription guidelines |  |  |  |  |  |
| Existence of tools to facilitate deprescription |  |  |  |  |  |
| Communication with other physicians from other specialties |  |  |  |  |  |
| Collaboration with physicians from other specialties |  |  |  |  |  |
| Time consumption |  |  |  |  |  |
| Benefit of medication |  |  |  |  |  |
| Medication risks |  |  |  |  |  |

1. In your opinion are there other factors that can influence deprescription?

- Yes
- No
  1. What are these other factors that can influence deprescription?

1. Do you have any additional comments regarding deprescription?
